# GV‐971 attenuates α‐Synuclein aggregation and related pathology

**DOI:** 10.1111/cns.14393

**Published:** 2023-08-10

**Authors:** Zhenwei Yu, Ying Yang, Robin Barry Chan, Min Shi, Tessandra Stewart, Yang Huang, Zongran Liu, Guoyu Lan, Lifu Sheng, Chen Tian, Dishun Yang, Jing Zhang

**Affiliations:** ^1^ Beijing Neurosurgical Institute Capital Medical University Beijing China; ^2^ Department of Pathology, The First Affiliated Hospital Zhejiang University School of Medicine Hangzhou China; ^3^ National Health and Disease Human Brain Tissue Resource Center Zhejiang University Hangzhou China; ^4^ AliveX Biotech Shanghai China; ^5^ Department of Pathology University of Washington School of Medicine Seattle Washington USA; ^6^ Department of Pathology Peking University Health Science Center and Third Hospital Beijing China

**Keywords:** extracellular vesicle, GV‐971, Synucleinopathy, α‐Synuclein aggregation

## Abstract

**Rationale:**

Synucleinopathies, including Parkinson's disease (PD), multiple system atrophy (MSA), and dementia with Lewy bodies (DLB), share a distinct pathological feature, that is, a widespread accumulation of α‐synuclein (α‐syn) in the brain. There is a significant clinical unmet need for disease‐modifying treatments for synucleinopathies. Recently, a seaweed‐derived mixture of oligosaccharides sodium oligomannate, GV‐971, was approved for Phase 2 clinical trials for PD. This study aimed to further evaluate the therapeutic effects of GV‐971 on synucleinopathies using cellular and animal models and explore its associated molecular mechanisms.

**Methods:**

α‐Syn aggregation was assessed, in vitro and ex vivo, by ThT assay. A dopaminergic neuron cell line, *Prnp‐SNCA* ^A53T^ mice, and brain slices from PD and DLB patients were used to determine the efficacy of GV‐971 in ameliorating α‐syn pathology. Measurements of motor functions, including pole, cylinder, and rotarod tests, were conducted on *Prnp‐SNCA* ^A53T^ mice 4 weeks after intragastric administration of GV‐971 (200 mg day^−1^ kg^−1^).

**Results:**

GV‐971 effectively prevented α‐syn aggregation and even disassembled pre‐aggregated α‐syn fibrils, in vitro and ex vivo*.* In addition, GV‐971 was able to rescue α‐syn‐induced neuronal damage and reduced release of extracellular vesicles (EVs), likely via modulating Alix expression. In the *Prnp‐SNCA* ^A53T^ mouse model, when treated at the age of 5 months, GV‐971 significantly decreased α‐syn deposition in the cortex, midbrain, and cerebellum regions, along with ameliorating the motor dysfunctions.

**Conclusions:**

Our results indicate that GV‐971, when administered at a relatively early stage of the disease process, significantly reduced α‐syn accumulation and aggregation in *Prnp‐SNCA* ^A53T^ mice. Furthermore, GV‐971 corrected α‐syn‐induced inhibition of EVs release in neurons, contributing to neuronal protection. Future studies are needed to further assess GV‐971 as a promising disease‐modifying therapy for PD and other synucleinopathies.

## Graphical abstract

GV‐971 inhibits α‐synuclein aggregation and suppresses α‐syn‐induced EV dysfunctional via downregulation of *Alix*.

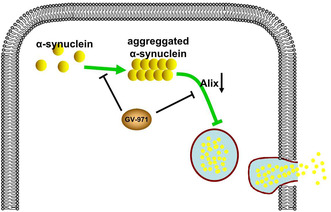

## 1 INTRODUCTION

Parkinson's disease (PD), multiple system atrophy (MSA), and dementia with Lewy body (DLB) are broadly defined as synucleinopathies.^1^ PD, the most common type of synucleinopathy, is clinically characterized by motor symptoms resulting largely from the degeneration of dopaminergic neurons in the *substantia nigra pars compacta* (SNc).^2^, ^3^ Synucleinopathies share a common pathological characteristic by the accumulation of α‐synuclein (α‐syn) aggregates in the brain, a process believed to be central to the pathogenesis of these diseases. Aggregated α‐syn includes oligomers, protofibrils, and fibrils, among which the soluble oligomers and protofibrils are believed to be the most toxic species to the central nervous system (CNS).^4^, ^5^

Currently, there is no cure for PD or any other synucleinopathies, but medications, surgeries, and a few complementary and supportive treatments are available to help to relieve the symptoms, with dopamine replacement being the most common first‐line therapy for PD patients.^6^ While dopamine replacement is associated with significant improvements in motor functions, significant numbers of PD patients develop resistance to its beneficial effect as the disease progresses.^7^ In some patients with advanced PD who have unstable medication (levodopa) responses, deep brain stimulation may provide additional benefit for PD symptoms; but again, the treatment does not prevent the disease from progressing. For MSA and DLB, treatment options are even fewer. Thus, new disease‐modifying therapies for synucleinopathies are critically needed.

Reduction of α‐syn aggregations in the brain is a key mechanism of several disease‐modifying therapies that are currently under intensive investigation. For example, prasinezumab and BIIB054, two monoclonal antibodies targeting aggregated forms of α‐syn, are reported to induce clearance of α‐syn, reduce α‐syn accumulation and spreading, and protect against motor deficits.^8^, ^9^, ^10^ Several small molecules, including anle138b, gallic acid, ginsenoside Rb1, NPT100‐18A, and NPT200‐11,^11^, ^12^, ^13^, ^14^ have been investigated for their ability to inhibit α‐syn aggregation. Small interfering RNAs (siRNAs),^15^ short hairpin RNAs (shRNAs),^16^ and microRNAs,^17^ which could downregulate or silence α‐syn expression, have also been tested for potential neuroprotection. Despite significant research into these potential targets, the progress in developing new PD disease‐modifying therapies is obviously too slow to meet the critical need of effectively treating PD or related synucleinopathies.

In 2019, a therapeutic candidate containing a mixture of oligosaccharides extracted from brown algae (Sodium Oligomannate, GV‐971) received conditional approval from the China National Medical Products Administration for the treatment of mild‐to‐moderate Alzheimer's disease (AD). GV‐971 was found to improve cognitive behavior of the 5xFAD AD mice in a mechanism associated with the restoration of the abnormal gut microbiome pattern and reduction of neuroinflammation,^18^ a result reproduced recently by an independent study conducted in United States (https://cslide.ctimeetingtech.com/global_storage/media/content/adpd23/ADPD23_‐_Posters_for_website_Mar_29.pdf). In addition, in vitro experiments demonstrated that GV‐971 could inhibit the aggregation of Aβ and the Aβ‐induced cellular damage.^19^ Jiang et al. found that GV‐971 inhibited the conformational transition from α‐helix to β‐sheet and the hydrophobic collapse of the Aβ42 monomer,^20^ which is also important in α‐syn aggregation.^21^ As GV‐971 was shown to be safe and well tolerated in both patients and healthy volunteers in AD‐related clinical studies (https://clinicaltrials.gov/ct2/show/NCT05058040), the US Food and Drug Administration recently approved a global multicenter phase II clinical trial of GV‐971 in PD (investigational new drug (IND) 159315), though, to date, there is no information on this trial available in ClinicalTrials.gov. Nevertheless, the therapeutic effects and potential mechanisms of GV‐971 in PD treatment remain to be fully explored.

In this study, we investigated the effects of GV‐971 on α‐syn aggregation at three levels, that is, in vitro using a protein aggregation assay and a dopaminergic neuronal cell line, ex vivo using PD and DLB brain slices, and in vivo using a commonly used PD mouse model, *Prnp‐SNCA* ^A53T^. We also explored the effects of GV‐971 on neuronal extracellular vesicle (EV) secretion that was disrupted by α‐syn overexpression. Lastly, we tested if GV‐971 had any therapeutic benefits on the motor deficits typically seen in *Prnp‐SNCA* ^A53T^ mice.

## 2 MATERIALS AND METHODS

### 2.1 Human samples

This study was approved by the Institutional Review Board at the University of Washington (IRB: STUDY00003047). Autopsy materials used in this study were obtained from the University of Washington Neuropathology Core, which is supported by the Alzheimer's Disease Research Center (AG05136) and the Adult Changes in Thought Study (AG006781). The brain tissues were collected from donors who had provided written informed consent for a brain autopsy and permitted their clinical information for research purposes. A summary of the demographics and clinical data of the participants is provided in Table S1. Brain specimens of the *substantia nigra* (SN) from three PD patients, three DLB patients, and three controls were included in this study. Formalin‐fixed and paraffin‐embedded specimens were sectioned at 7 μm thickness and used for immunofluorescence staining.

### 2.2 Immunofluorescence/fluorescence staining

To assess α‐syn expression and the colocalization of α‐syn and Alix in the brain, the postmortem human brain tissues and the left hemisphere of the mouse brain were embedded in OCT and sectioned at 8‐μm thickness. The sections were fixed with 4% PFA for 30 min, followed by 30 min of blocking with 3% bovine serum albumin (BSA) in PBS. For the brain sections from DLB patients, 2 mM GV‐971 was administered to the brain slices for 3‐day incubation at 37°C before staining. Primary antibodies and ThT reagent were used as follows: rabbit anti‐α‐syn (ab138501, Abcam, 1:1000); mouse anti‐α‐syn (610787, BD Biosciences, 1:500); rabbit anti‐α‐syn aggregate (ab209538, Abcam, 1:1000); and rabbit anti‐Alix (ABC40, Millipore, 1:1000), and 0.05% ThT (596200, Sigma Aldrich). The primary antibodies were incubated overnight at 4°C, followed by incubation of corresponding secondary antibodies that were conjugated with Alexa Fluor 488 or 647 (Abcam; 1:1000) at room temperature for 1 h. DAPI (0.5 μg/mL) was used for nuclear staining. Images of randomly selected fields (*n* ≥ 5) were acquired using an LSM 710 confocal microscope (Carl Zeiss) and quantified by using ImageJ software (NIH).

### 2.3 Western blotting

Western blotting was performed following the standard protocol. Samples were solubilized with Laemmli buffer and separated on SDS–PAGE gels or native gels before transferring to a PVDF membrane. The assay was carried out using α‐syn primary antibodies (rabbit anti‐α‐syn, ab138501, Abcam, 1:10000; rabbit anti‐α‐syn aggregate, ab209538, Abcam, 1:10000) to detect α‐syn expression in mouse brain homogenates and a dopaminergic neuron cell line (Mn9D). Other primary antibodies included anti‐beta‐actin (1:1000, ab8226; Abcam) and anti‐Alix (ABC40, 1:100). The PVDF membranes were incubated with primary antibodies overnight at 4°C, followed by three washes using TBST. Then, the secondary antibodies were applied to the membranes for 1 h at room temperature. The secondary antibodies include horseradish peroxidase‐goat anti‐rabbit IgG (ZB‐2301, ZSGB‐BIO, 1:5000) and horseradish peroxidase‐goat anti‐mouse IgG (ZB‐2305, ZSGB‐BIO, 1:5000). Images were visualized using an ECL substrate kit (WBULS0100, Millipore) and captured with a ChemiDoc MP imaging system (Bio‐Rad). The protein bands were quantified by using ImageJ software (NIH). All raw images were presented in Figure S2.

### 2.4 Thioflavin T (ThT) protein aggregation assay

The ThT assay was used to detect the dynamic aggregation of α‐syn and the rescue effect induced by GV‐971. Recombinant α‐syn was purchased from SinoBiological Inc (12093‐HNAE). ThT was purchased from Sigma Aldrich (596200). Briefly, all reagents were dissolved in PBS (pH 7.4) at final concentrations of α‐syn 1 mg/mL, ThT 10 μM, and GV‐971 0–200 μg/mL. Experiments were carried out in 96‐well black/bottom clear plates. Time traces were recorded using a BMG CLARIOstar plate reader with λ_exc_ 450 nm and λ_em_ 480 nm at 37°C for 8 days. The plate was shaken for 5 min before reading followed by a 10 min standby for each cycle, and 480 cycles/readings were recorded (*n* = 3 for each group). For the test of GV‐971 effect on pre‐aggregated α‐syn, 100 μL monomeric α‐syn (1 mg/mL) with 10 μM ThT was shaken for 120 h to generate aggregates, and then, the GV‐971 was added to the solution at a concentration of 100 μg/mL.

### 2.5 Transmission electron microscopy imaging

Mn9D cells transfected with an empty vector or pCMV3‐SNCA (human) α‐syn overexpression plasmid (HG12093‐CH, SinoBiological) with or without 100 μg/mL GV‐971 treatment were pelleted by centrifugation at 2000*g* for 10 min and fixed using 2.5% (v/v) glutaraldehyde overnight at 4°C. The cells were carefully washed three times with PBS (pH 7.4) and then postfixed for 2 h in 1% (w/v) OsO4 at 4°C. The samples were dehydrated with an acetone gradient from 30% to 100%, followed by 50%, 67%, 75% (v/v) araldite in acetone, and then embedded in araldite. The samples were sectioned at 50 nm thickness, stained with saturated uranyl citrate and 0.2% (w/v, pH 11) lead acetate for 30 min at room temperature, and then washed three times with ddH_2_O. Sections were then mounted onto transmission electron microscopy (TEM) copper grids. For the α‐syn aggregates, samples were mixed 1:1 with 5% (v/v) glutaraldehyde overnight at 4°C for fixation. Then, the mixtures were layered onto copper grids and allowed to dry for 30 min. The samples were then stained with saturated uranyl citrate and 0.2% (w/v, pH 11) lead acetate for 30 min and washed three times with ddH_2_O before imaging on a HEM‐1400 PLUS microscope (JEOL).

### 2.6 Cell viability assay

Cell viability was assessed by MTS assay from Progema (G3582, USA). Mn9D cells (5 × 10^3^) were counted and seeded into each well of a 96‐well plate. The cells were then transfected with an empty vector or α‐syn overexpression plasmid (pCMV3‐SNCA) using the jetPRIME transfection reagent (Polyplus transfection). The culture medium was replaced 6 h after transfection, and GV‐971 with various concentrations (0–200 μg/mL) was added to the medium. Thirty‐six hours after transfection, the culture medium was replaced again with MTS solution diluted in DMEM medium at a final concentration of 0.2 mg/mL for 2 h before plate reading. The absorbance at 490 nm of each well was recorded on a BMG CLARIOstar plate reader.

### 2.7 Flow cytometry and propidium iodide (PI) staining

Mn9D cells (5 × 10^3^) with different treatments were pelleted by centrifugation at 200*g* and then washed twice with PBS (pH 7.4). The PI staining kit was from Sangon Biotech. A 5 μL PI reagent was added to a 95 μL cell suspension and incubated for 30 min. Then, the samples were transferred to a 5 mL round bottom test tube (352054, Falcon) for flow cytometric analysis on a Beckman CytoFLEX S. The gating strategy was presented in Figure S1.

### 2.8 EV nanoparticle tracking analysis

The culture medium of cells under different treatments was centrifuged at 1000*g* for 10 min to eliminate cell debris. The supernatant was collected and diluted 1:10 in PBS (pH 7.4) for the nanoparticle tracking analysis. A ZetaView (Particle Metrix) instrument was used for particle quantification in scatter mode following the manufacturer's protocol.

### 2.9 Quantitative PCR analysis

Total RNA of the Mn9D cells was extracted using a RNAsimple total RNA isolation kit (DP419, Tiangen). Then, the RNA was reverse‐transcribed to cDNA using the Goscript Reverse Transcription kit (A5000, Promega). The qPCR was carried out using SYB Green master mix (A25742, Life Technology) and ABI 7500 real‐time PCR system (Applied Biosystems). The mRNA levels of *Alix* were normalized to those of *GAPDH* levels with 2^−ΔΔCT^ method. The primer sequences of *Alix* and *GAPDH* are as follows: *Alix* forward primer: GCCCGTCAACAGTAGTTGAAT; *Alix* reverse primer: CCCGGAGAAGTACTGGTGTC; *GAPDH* forward primer: CGGAGTCAACGGATTTGGTCGTAT; *GAPDH* reverse primer: AGCCTTCTCCATGGTGGTGAAGAC.

### 2.10 Immunohistochemistry (IHC) staining

The right hemisphere of mouse brain tissue was fixed with 4% PFA and paraffin‐embedded. Sodium citrate buffer was used for antigen retrieval, and endogenous peroxidase was blocked by incubating in 3% hydrogen peroxide for 30 min. After washing three times with PBS, the sections were blocked using 3% BSA in PBS at room temperature for 30 min. For proteinase K (PK)‐resistant protein detection, the sections were preincubated with PK (Gibco BRL, Gaithersburg, MD; 50 μg/mL) in buffer containing 10 mM Tris–HCl, pH 7.8, 100 mM NaCl, and 0.1% NP40 at 37°C for 30 min. Afterward, the sections were washed three times with PBS. The primary antibodies (rabbit anti‐α‐syn, ab138501, Abcam, 1:1000 for normal α‐syn detection; rabbit anti‐α‐syn aggregate, ab209538, Abcam, 1:1000 for PK‐resistant α‐syn detection) were then added to the slices for overnight incubation at 4°C. The IHC results were visualized by using a horseradish catalase DAB color kit (Sangon). Finally, the sections were imaged from five randomly selected fields using an Olympus IX51 microscope.

### 2.11 Animals

*Prnp‐SNCA* ^A53T^ transgenic mice overexpressing human A53T mutant α‐syn under the control of the prion protein (prnp) promoter^22^ were purchased from Nanjing BioMedical Research Institute and used as a PD model in this study. Wild‐type (WT) C57/BL6 mice were used as controls. Only male mice were used in the following animal experiments. The mice were maintained and handled with the approval of the Institutional Review Board for Laboratory Animal Care and fed in a barrier environment in the Department of Laboratory Animal Science, Peking University Health Science Center, with a 12‐h light/dark cycle and free access to food and water.

GV‐971, a mixture of oligosaccharides extracted from brown algae, was manufactured by Shanghai Green Valley Pharmaceutical Co., Ltd. GV‐971 was dissolved in PBS (pH 7.4) at a concentration of 50 mg/mL. The drug was intragastrically administered at a dose of 200 mg day^−1^ kg^−1^ body weight daily for 4 weeks (*n* = 5), starting at 5 months of age, when no overt movement disorder is seen in the brain.^22^ The dose of GV‐971 applied was in accordance with the clinical dose for AD patients. The same volume of saline was used as the vehicle control treatment (*n* = 5 ~ 7). After GV‐971 administration, behavior tests, including the pole test, rotarod test, and cylinder test, were performed to monitor the alterations of movement abilities. When all behavior tests were finished, the animals were sacrificed for brain tissue collections.

### 2.12 Pole test

The pole test is a useful method to evaluate striatal dopamine depletion‐induced movement disorders.^23^ After 4 weeks of GV‐971 administration, the experiment was conducted as described previously with minor modifications. Briefly, the mice were head‐upward placed on the top of a vertically placed pole (40 cm in height and 1 cm in diameter). The time until all the legs of the mouse touched the floor was recorded. All time durations longer than 120 s or the mice falling from the pole were recorded as 120 s. Two training tests were performed 4 days ahead of the real measurements, and the average duration of three repeated tests was recorded as the performance of each mouse.

### 2.13 Rotarod test

The rotarod test is another widely used behavior test to evaluate the motor coordination, balance, and ataxia.^24^, ^25^ A 3‐cm diameter rotating rod starting with a rotation speed of 5 rpm was used in this test. The accelerating rotation speed ended at 15 rpm, and the latency to fall from the rod in three consecutive trials was recorded, with a cutoff time of 2 min (*n* = 5 for each group). The training test was conducted 4 days before the experiments, allowing the mice to get accustomed to the apparatus.

### 2.14 Cylinder test

This test was used to assess limb use frequency and asymmetry.^26^ Briefly, the mice were placed in a transparent glass cylinder (20 cm in diameter, 30 cm in height) for 3 min. The whole process was videoed, and the number of left/right forelimb contacts to the cylinder wall and total standing time were recorded (*n* = 5 for each group).

### 2.15 Statistical analysis

All the statistical analysis presented in this study was performed using Prism 9 (GraphPad Software). Data normality was tested using Kolmogorov–Smirnov test. For the data not normally distributed, differences between two groups were compared with the Mann–Whitney *U*‐test. One‐way ANOVA followed by Tukey test was used for three groups comparisons. *p* < 0.05 was considered statistically significant.

## 3 RESULTS

### 3.1 GV‐971 prevents α‐syn aggregation in vitro and ex vivo

**FIGURE 1:**
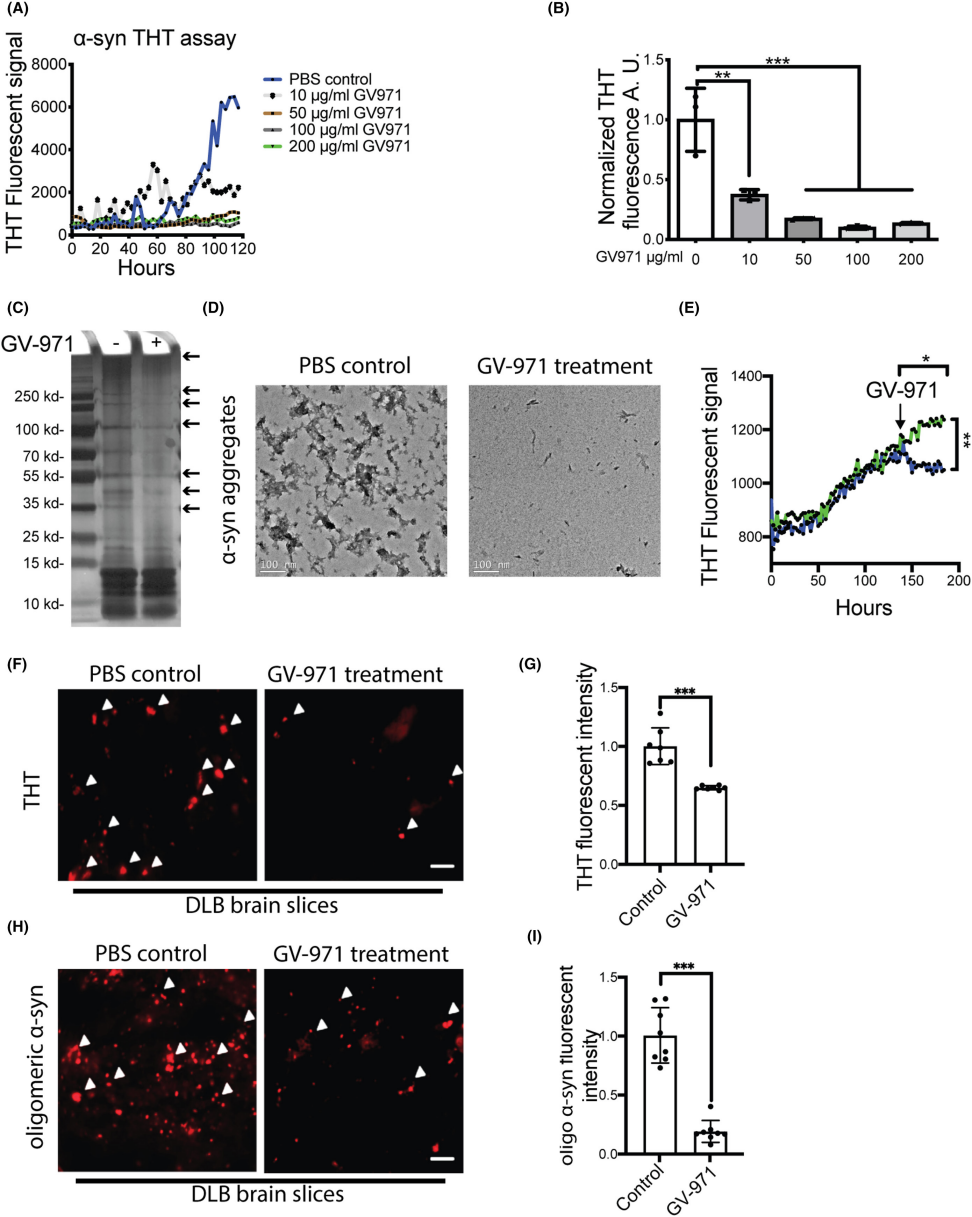
GV‐971 prevents α‐synuclein aggregation in vitro and ex vivo. (A) Dose response (0–200 μg/mL of GV‐971) of α‐syn aggregation over 120 h by ThT assay. (B) Reduction in relative ThT fluorescence signals at 120 h time point following exposure to 10–200 μg/mL GV‐971 treatment. Results are presented as mean ± SEM. **p < 0.01, ***p < 0.001. (C) Silver staining of the α‐syn aggregates with or without 100 μg/mL GV‐971 treatment. (D) TEM imaging showing disruption of α‐syn aggregates with exposure to 100 μg/mL GV‐971. (E) ThT assay demonstrating disaggregation of preformed α‐syn aggregates by GV‐971 treatment. *p < 0.05, **p < 0.01. (F, G) ThT fluorescence staining of brain slices from the cortex of DLB patients to measure the disaggregation of pathological aggregates through GV‐971 incubation. Scale bar = 20 μm, ***p < 0.001. (H, I) Oligomeric α‐syn staining of brain slices from the cortex of DLB patients to measure the disaggregation of pathological aggregates through GV‐971 incubation. Scale bar = 20 μm, ***p < 0.001. Experiments were performed three times with *n* ≥ 3 technical replicates each time. α‐syn, α‐synuclein.

To determine whether GV‐971 inhibits the aggregation of α‐syn directly, we performed a ThT protein aggregation assay with GV‐971 at concentrations ranging from 10 to 200 μg/mL, according to the effective concentrations applied in AD and Aβ studies previously.^19^, ^20^ A relatively muted ThT signal was observed in the GV‐971 treatment group compared to the PBS group even after a lag time of 5 days, confirming the inhibitory effect of GV‐971 on α‐syn aggregation in vitro (Figure 1A,B). Silver staining also revealed that 100 μg/mL GV‐971 reduced the formation of oligomeric α‐syn of sizes ranging from 60 kD to 200 kD (Figure 1C). The biophysical effects of GV‐971 on α‐syn aggregation were also assessed by TEM, showing that GV‐971 treatment strongly reduced the formation of α‐syn aggregates induced by 5 days of continuous shaking (Figure 1D).

Next, we added GV‐971 to a solution of α‐syn fibrils that had been accumulating for almost 150 h, and showed that GV‐971 could also reverse the accumulation of pre‐formed α‐syn aggregates to some extent (Figure 1E). This led us to explore whether GV‐971 could decrease preformed α‐syn aggregates in patient‐derived brain tissues directly. The results showed that GV‐971‐treated brain slices from three DLB patients exhibited significantly reduced ThT‐positive plaques (Figure 1F,G) and oligomeric α‐syn (Figure 1H,I).

### 3.2 GV‐971 rescues α‐syn‐induced neuronal damage in a cellular model

**FIGURE 2:**
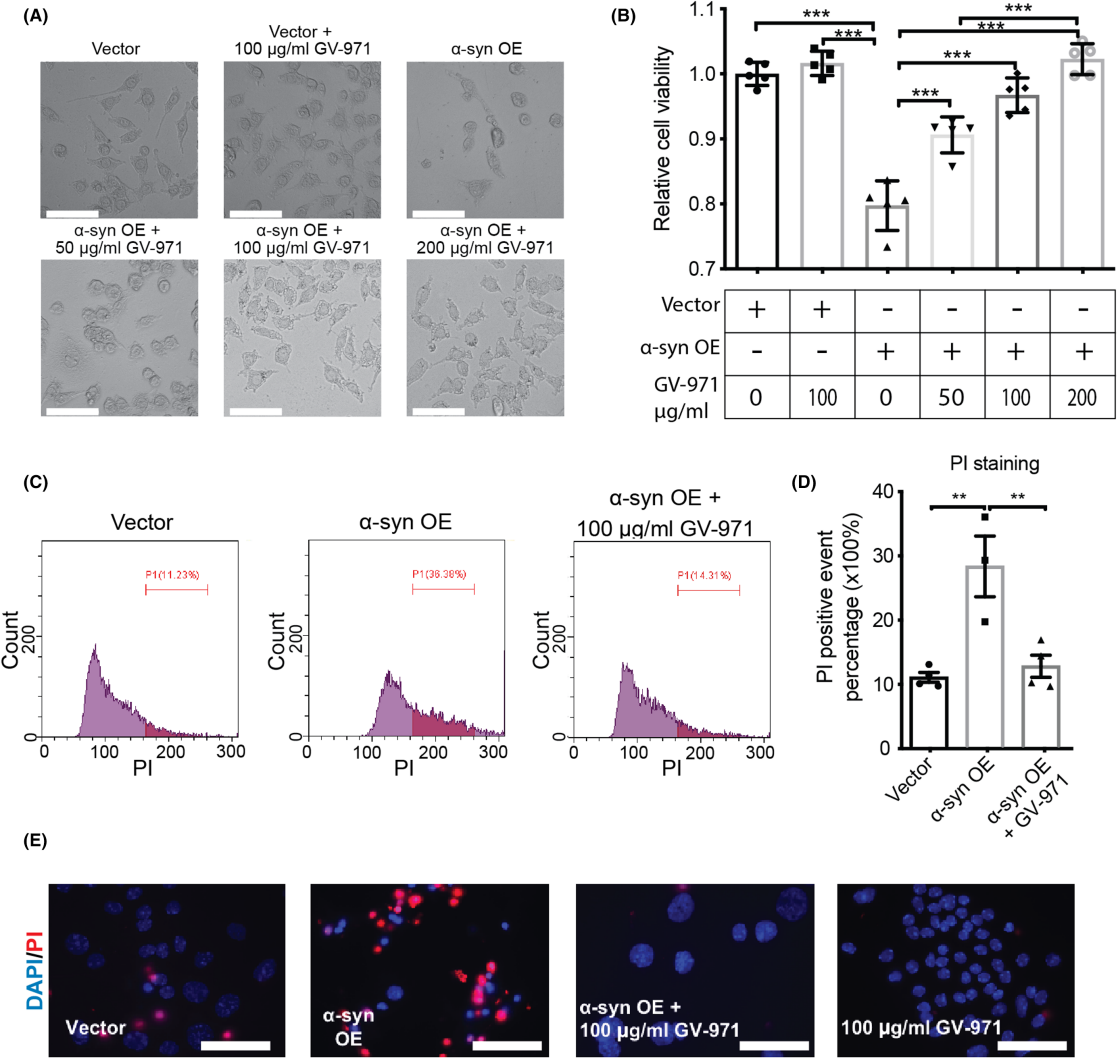
GV‐971 rescues α‐syn‐induced neuronal death. (A) The morphology of Mn9D cells overexpressing α‐syn or empty vector with 0–200 μg/mL GV‐971 treatment. Scale bar: 75 μm. (B) MTS assay measuring the viability of Mn9D cell overexpressing α‐syn or empty vector in the presence of 0–200 g/mL GV‐971. ***p < 0.001. (C, D) Flow cytometry analysis with PI staining to measure the cell death in cells overexpressing α‐syn or empty vector with 100 μg/mL GV‐971 treatment. *p < 0.05, **p < 0.01. (E) Fluorescence imaging of PI staining to measure the cell death when overexpressing α‐syn or empty vector with 100 μg/mL GV‐971 treatment. Scale bar: 100 μm. Experiments were performed three times with *n* ≥ 3 technical replicates each time. α‐syn, α‐synuclein; OE, overexpression; PI, propidium iodide.

To investigate the effects of GV‐971 on the neuronal function, α‐syn was overexpressed in Mn9D cells, a cell line derived from dopaminergic neurons, followed by GV‐971 incubation for 24 h at different concentrations. As expected, the results showed that α‐syn overexpression induced a significant morphological change in the Mn9D cells (Figure 2A), similar to those in previous reports,^27^ and a reduction in cell viability as determined by MTS assay (Figure 2B). Importantly, both phenotypes could be rescued by 50–200 μg/mL GV‐971 treatments in a concentration‐dependent manner (Figure 2A,B). PI assay is widely used for the evaluation of cell viability and death. The presence of α‐syn induced neuronal damage and the effect of GV‐917 on this phenomenon were also validated by PI staining followed by flow cytometry analysis (Figure 2C,D) and immunofluorescence imaging (Figure 2E). α‐Syn overexpression significantly increased the cell death of Mn9D cells, which can be rescued substantially with the treatment of GV‐971.

### 3.3 α‐Syn reduces EV generation and release in cultured neurons

**FIGURE 3:**
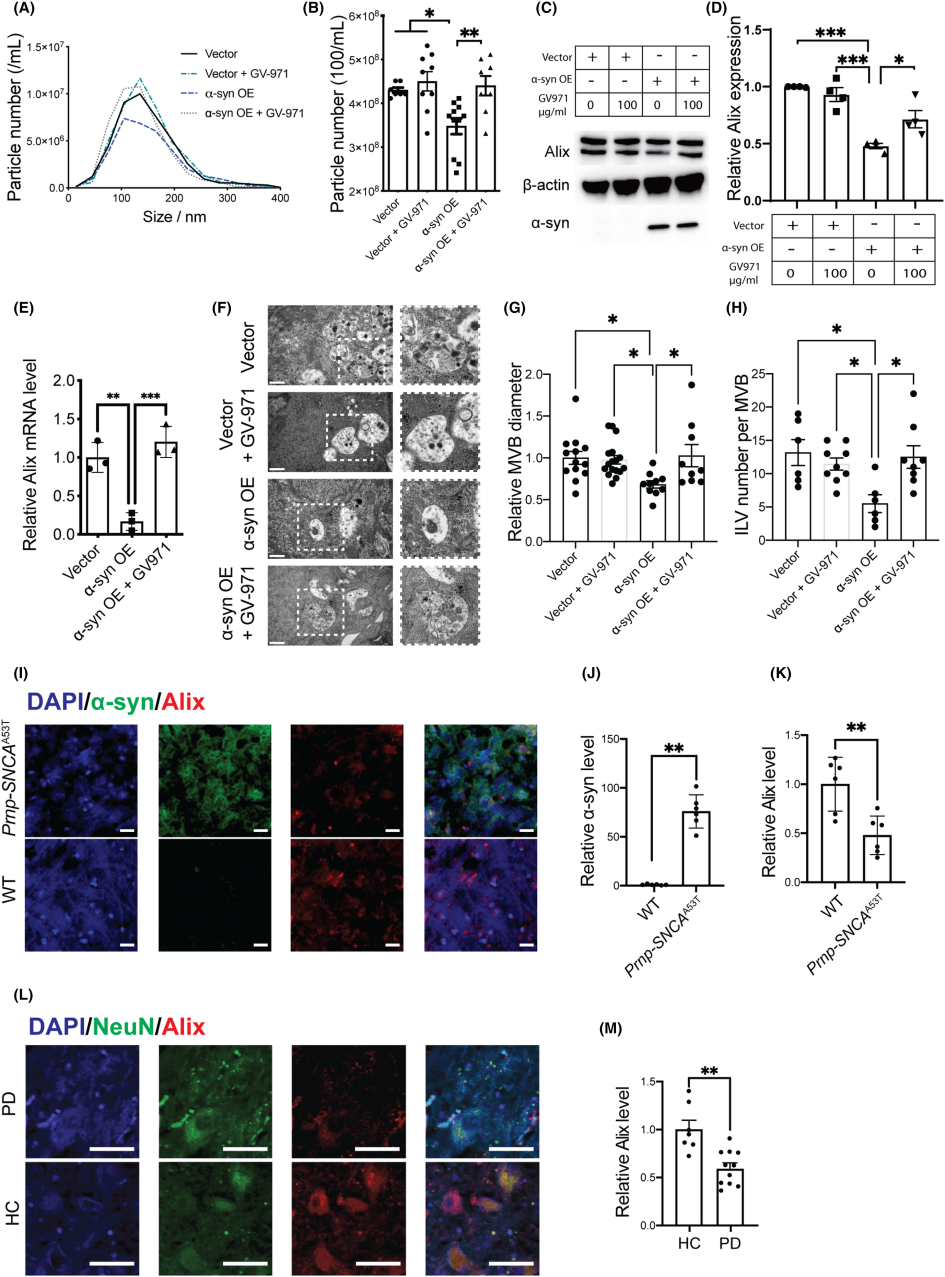
GV‐971 rescues α‐syn‐induced EV generation and release in neurons. (A, B) NTA analysis measuring the EV concentrations in the conditioned media of Mn9D cells overexpressing empty vector or α‐syn with GV‐971 treatment at 100 μg/mL. *p < 0.05, **p < 0.01. (C, D) Western blot analysis of Alix levels in Mn9D cells overexpressing empty vector or α‐syn with 0–100 μg/mL GV‐971 treatment. (E) qPCR analysis of *Alix* mRNA levels in Mn9D cells overexpressing empty vector or α‐syn with 100 μg/mL GV‐971 treatment. **p < 0.01, ***p < 0.001. (F) TEM images of MVBs in Mn9D cells overexpressing empty vector or α‐syn with 100 μg/mL GV‐971 treatment. Scale bar: 400 nm. (G, H) The relative MVB diameter and ILV numbers per MVB in Mn9D cells overexpressing empty vector or α‐syn with 100 μg/mL GV‐971 treatment. *p < 0.05. (I) Immunofluorescence staining of α‐syn and Alix in the brain slices from the midbrain of WT and *Prnp‐SNCA* ^A53T^ mice. Quantifications of α‐syn (J) and Alix (K) intensities. Scale bar = 20 μm, ** *p* < 0.01. (L) Immunofluorescence analysis of NeuN and Alix expression in the SN region of PD patients and HCs. Scale bar = 20 μm. (M) Quantification of Alix intensities in neurons. **p < 0.01. Experiments were performed three times with *n* ≥ 3 technical replicates each time. α‐syn, α‐synuclein; HC, healthy control; OE, overexpression; PD, Parkinson's disease; WT, wild type.

The secretion of EVs, including exosomes, is an important mechanism by which toxic proteins are cleared from cells.^28^, ^29^ Through NTA analysis of EV concentrations in cell culture medium, it was shown that α‐syn overexpression in Mn9D significantly reduced the release of EVs, an effect that was readily reversed by GV‐971 treatment (Figure 3A,B).

To probe the potential regulators of changes in α‐syn regulated EVs secretion, the level of Alix, a key protein associated with ESCRT‐III that regulates intraluminal vesicle (ILV) budding, MVB formation, and exosome release,^30^, ^31^ was evaluated. As shown in Figure 3C,D, overexpression of α‐syn significantly reduced the protein and mRNA levels of Alix, and the downregulation of Alix was rescued by GV‐971 treatment. At the morphological level, TEM analysis showed that the average ILV numbers in MVBs, as well as the MVB diameters, were decreased when α‐syn was overexpressed in Mn9D cells, and 100 μg/mL GV‐971 treatment for 24 h rescued the α‐syn‐induced MVB dysfunction (Figure 3E–G). No significant changes in ILV formation or MVB morphology were found in Mn9D cells with GV‐971 treatment alone compared to the vector control group.

To establish the relevance of this EV/Alix‐related mechanism in mouse and human brains, we first studied Alix expression in the brains of WT and *Prnp‐SNCA* ^A53T^ mice. Immunofluorescence imaging (Figure 3H) showed that α‐syn expression in *Prnp‐SNCA* ^A53T^ mice was significantly higher than that in WT mice (Figure 3I), while the Alix levels were significantly decreased accordingly (Figure 3J). Next, we verified the decrease of Alix by immunofluorescence staining in the SN slices of PD patients compared to healthy controls (Figure 3K,L).

### 3.4 GV‐971 decreases α‐syn level in the brains of *Prnp‐SNCA* ^A53T^ mice

To validate the results observed in vitro and ex vivo, we studied the effect of GV‐971 on α‐syn levels in *Prnp‐SNCA* ^A53T^ mice, which specifically overexpress human α‐syn in neurons under the control of the prion protein promotor. GV‐971 was intragastrically administered daily at a dose of 200 mg/kg for 4 weeks in *Prnp‐SNCA* ^A53T^ mice at 20 weeks of age (Figure 5A), that is, at a stage prior to motor symptoms are typically observed in these mice.^22^

**FIGURE 4:**
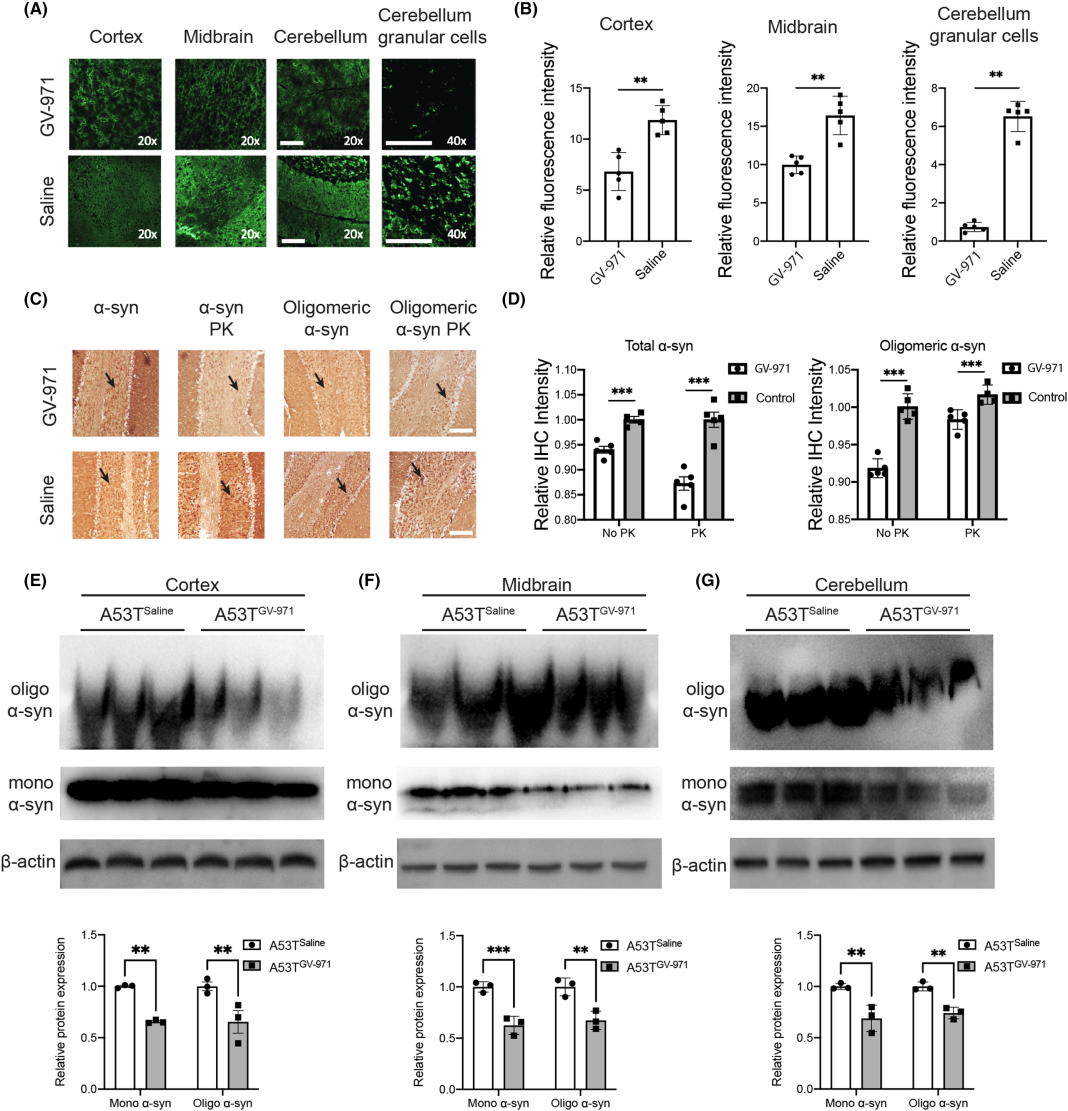
GV‐971 decreases α‐syn level in the brains of *Prnp‐SNCA* ^A53T^ mice. (A) Immunofluorescence analysis of α‐syn expression in the cortex, midbrain, and cerebellum of *Prnp‐SNCA* ^A53T^ with or without GV‐971 administration, (B) and the corresponding quantifications. **p < 0.01. Scale bar: 150 μm. (C) Immunohistochemistry analysis of total and oligomeric α‐syn levels with or without PK treatment in the cerebellum of GV‐971 or saline administered *Prnp‐SNCA* ^A53T^ PD mice with or without PK treatment. Scale bar: 150 μm. (D) The quantifications of total and oligomeric α‐syn levels of the immunohistochemistry images. ***p < 0.001. (E–G) Western blot analysis and quantifications of total and oligomeric α‐syn levels in the cortex, midbrain, and cerebellum of GV‐971 or saline administered *Prnp‐SNCA* ^A53T^ mice and the quantifications. **p < 0.01, ***p < 0.001. Experiments were performed three times with *n* ≥ 3 technical replicates each time. α‐syn, α‐synuclein; mono, monomeric; oligo, oligomeric; PK, proteinase K.

Immunofluorescence imaging revealed that α‐syn expression in the cortex, midbrain, and cerebellum was significantly reduced after 4 weeks of GV‐971 administration (Figure 4A,B). Notably, GV‐971 significantly reduced the deposition of total and oligomeric α‐syn in cerebellar granule cells, which plays an essential role in motor learning.^32^ Immunohistochemistry analysis also revealed that GV‐971 reduced the deposition of both total and oligomeric α‐syn in the cerebellum of PD mice under normal as well as PK‐resistant conditions (Figure 4C,D). The expression of α‐syn species across the brains of PD mice was also determined by western blotting. The levels of both total and oligomeric α‐syn in the GV‐971‐treated group were significantly reduced in the cortex (Figure 4E), midbrain (Figure 4F), and cerebellum (Figure 4G) regions.

### 3.5 GV‐971 improves the motor function of *Prnp‐SNCA* ^A53T^ mice

**FIGURE 5:**
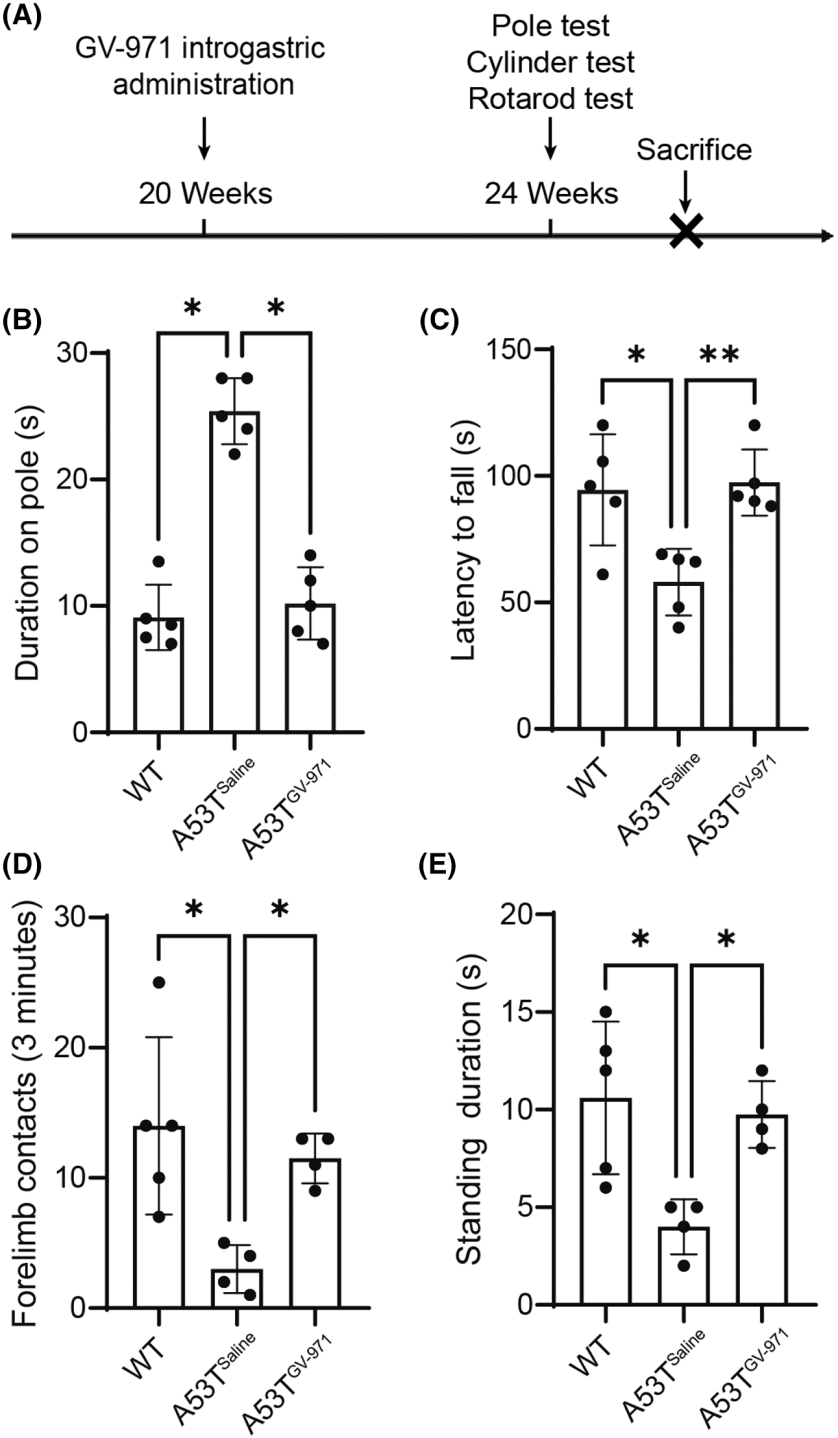
GV‐971 improves the motor function of *Prnp‐SNCA* ^A53T^ mice. (A) Schematic representation of the GV‐971 intragastric administration and motor behavior tests of the *Prnp‐SNCA* ^A53T^ mice. (B) The time taken for the mice to land from the top for WT and *Prnp‐SNCA* ^A53T^ mice with or without GV‐971 administration in the pole test. *p < 0.05. (C) The latency to fall in the rotarod test for WT and *Prnp‐SNCA* ^A53T^ mice with or without GV‐971. *p < 0.05, **p < 0.01. (D) The contacts of forelimbs to the wall within 3 min for the WT and *Prnp‐SNCA* ^A53T^ mice with or without GV‐971 administration in the cylinder test. *p < 0.05. (E) The standing durations within 3 min for the WT and *Prnp‐SNCA* ^A53T^ mice with or without GV‐971 administration in the cylinder test. *p < 0.05. *n* = 4–5. A53T, *Prnp‐SNCA* ^A53T^ mice; WT, wild type.

To investigate the potential of GV‐971 to prevent PD‐related symptoms, motor function assessments, including the pole test, rotarod test, and cylinder test, were conducted to evaluate the beneficial effects of GV‐971 on the motor symptoms of *Prnp‐SNCA^A53T^* mice (Figure 5A). The tests showed that GV‐971‐treated *Prnp‐SNCA^A53T^* mice required less time to descend the pole (Figure 5B) and had fewer falls off the rod compared to saline‐treated control *Prnp‐SNCA^A53T^* mice (Figure 5C). Next, a cylinder test was conducted to evaluate the limb usage dysfunction. The GV‐971 treated *Prnp‐SNCA^A53T^* mice showed improved usage of forelimbs (Figure 5D) and a longer total standing time (Figure 5E) compared to the saline‐treated *Prnp‐SNCA^A53T^* mice.

## 4 DISCUSSION

In this study, we found that GV‐971 has a potent protective effect on α‐syn‐induced neuronal damage, in vitro and in vivo, with at least two potential mechanisms involved, specifically, (1) attenuating oligomeric α‐syn aggregation and accumulation, and (2) promoting clearance or secretion of aggregated α‐syn from cells, at least in part, via EV release.

The first mechanism demonstrated in our study is that GV‐971 was capable of inhibiting α‐syn aggregation and disaggregating pre‐formed α‐syn aggregates. This protective mechanism was demonstrated using an in vitro synthetic α‐syn aggregation assay, as well as an ex vivo assay using DLB human tissue with α‐syn inclusions. Consistent with this observation, treatment of a dopaminergic neuronal cell line, Mn9D, that overexpresses WT α‐syn also reduced α‐syn aggregation while preventing cytotoxicity. The biophysical mechanisms by which GV‐971 inhibits α‐syn aggregation or disassembles pre‐aggregated α‐syn remain to be investigated. Molecular dynamic simulation demonstrates that the components of GV‐971 can effectively inhibit the conformational transition from α‐helix to β‐sheet and prevent the hydrophobic collapse of the Aβ42 monomer.^20^ It is likely that a similar mechanism is involved in preventing α‐syn aggregation. However, it is important to note that the mechanisms underlying the disassembly of pre‐aggregated α‐syn and prevention of α‐syn aggregation by GV‐971 may differ significantly. To provide a more comprehensive understanding of GV‐971's impact on α‐syn conformation and hydrophobic collapse, additional experiments such as thermodynamic characterization (e.g., differential scanning calorimetry, differential scanning fluorimetry) or structural characterization techniques could be employed in future investigations. These approaches would enable a direct examination of the effects of GV‐971 on α‐syn's secondary structure, stability, and hydrophobic interactions. Furthermore, investigating the interaction between GV‐971 and specific regions of α‐syn could help elucidate the molecular basis of GV‐971's inhibitory effects.

The second mechanism demonstrated in this study relates to the observation that GV‐971 restored EV formation and release from neuronal cell lines overexpressing α‐syn, with significantly reduced production of Alix at both mRNA and protein levels. Corresponding to the central role of Alix in the MVB formation, we observed that the diameter of MVBs, the number of ILVs within the MVBs, and exosome release in α‐syn‐overexpressing neurons were reduced significantly. However, these phenotypes were significantly corrected with GV‐971 treatment. Exosomes are normally formed as ILVs and stored within MVBs until MVBs fuse along the plasma membrane to release exosomes to the extracellular environment.^33^, ^34^ This process is essential for the proper function of the endolysosomal organelle system, which is in turn important for cellular regulation of lipid metabolism and protein recycling.^35^ It is conceivable that low Alix levels may trigger the blockage and corruption of the endolysosomal pathway, resulting in dysregulation of lipid composition that promotes the clustering and aggregation of known cargo such as α‐syn into its toxic oligomeric form. It is unclear whether GV‐971 directly regulates neuronal Alix expression or indirectly affects it through its interaction with other cellular components, such as inhibiting α‐syn aggregation, which itself may trigger lysosomal dysfunction.^36^ Nonetheless, it is likely that multiple corrective mechanisms are involved, resulting in a cumulative protective effect on the neuronal cells.

The accumulation of α‐syn in brain has been considered the most important pathogenesis in synucleinopathies, including PD.^2^ An important result of our study is that GV‐971 intragastric administration significantly improved the motor symptoms of *Prnp‐SNCA^A53T^* mice, along with a significant reduction in oligomeric α‐syn in several key brain regions. Of note, GV‐971 administration reduced both the monomeric and aggregated α‐syn levels in the brain of *Prnp‐SNCA* ^A53T^ mice. Although the α‐syn expression is at a significantly high level in *Prnp‐SNCA* ^A53T^ mice, it is crucial to carefully evaluate the potential loss‐of‐function effect of α‐syn induced by GV‐971, since α‐syn has several essential physiological functions, including a regulatory effect of immune functions of microglia.^37^ Even though GV‐971 administration alone did not affect the viability of dopaminergic neurons in vitro (Figure 2), further validations are needed in vivo and in other cell types in future investigations.

Another important question is whether GV‐971's effect was a direct interaction of the drug with the cells or targets of the CNS itself. As shown in a previous study, GV‐971 readily penetrated the blood–brain barrier,^38^ thus enabling the compound to physically interact with neuronal cells where α‐syn aggregation occurs. Of note, although the α‐syn aggregates of *Prnp‐SNCA^A53T^* mice were alleviated throughout the whole brain after 4 weeks of GV‐971 administration, the most prominent rescue effects occurred in the cerebellum granular cells, which play an important role in motor learning,^32^ and hence is likely one of the main reasons for improved motor performance in GV‐971‐treated *Prnp‐SNCA^A53T^* mice. In this study, we administered GV‐971 to *Prnp‐SNCA^A53T^* mice from 5 to 6 months of age. Although the pathological phenotype starts at 2–3 months of age in *Prnp‐SNCA^A53T^* mice, most of the motor dysfunctions are only revealed at 6 months of age.^22^, ^39^ Therefore, based on the design of the current study, we can only demonstrate that GV‐971 administration could prevent the development of PD at an early stage. Whether the drug has a similar protective efficacy at the late stage of PD remains to be studied.

As mentioned previously, over the past several years, numerous therapeutic compounds, including monoclonal antibodies^8^, ^9^, ^10^ and small molecules,^11^, ^12^, ^13^, ^14^ have been shown to reduce α‐syn aggregations in the brain, either through inhibition of α‐syn aggregation or increasing the clearance of accumulated α‐syn aggregates. However, these compounds have failed to advance beyond Phase I/II clinical trials, suggesting that while α‐syn aggregate reduction is important, it alone is insufficient to predict a strong therapeutic effect. To this end, it should be noted that, typically, orally administration of GV‐971 exerts a bioavailability about 4.2%–9.3%,^38^ and the drug concentration is even lower in the brain. At this point, we cannot directly compare the results of GV‐971 in vivo model that lasted a few months versus these in vitro experiments where much higher concentrations of GV‐971 were used in a much shorter period. In future studies, it would be critical to conduct additional investigation involving long‐term and low‐dosage GV‐971 treatment in vitro. Regardless, it is important to understand additional molecular mechanisms beyond anti‐α‐syn aggregation or disassembly of pre‐aggregated α‐syn. It was previously shown that GV‐971 alleviated AD pathology by restoring the abnormal gut microbiome pattern and reducing neuroinflammation.^18^ Like AD, abnormal gut microbiome population and neuroinflammation are also pathogenic features in PD.^40^, ^41^ These parallels suggest that the potential therapeutic effects of GV‐971 in PD may extend indeed beyond its direct impact on α‐syn and involve modulation of the gut microbiome and neuroinflammation, which should be investigated in future studies.

In conclusion, our investigation demonstrates that GV‐971 exhibited significant protective effects, in vitro, ex vivo, and in vivo, on the pathology related to α‐syn aggregation, and the mechanism of actions likely involve both disaggregating α‐syn and correcting α‐syn‐induced inhibition of neuronal exosome release. Considering the shared α‐syn pathology of PD with other synucleinopathies, including MSA and DLB, and given the paucity of disease‐modifying therapy for synucleinopathies, it is important to explore the therapeutic effects of GV‐971 in these other disease models in future studies.

## AUTHOR CONTRIBUTIONS

Zhenwei Yu, Ying Yang, and Jing Zhang contributed to the design of this study. Zhenwei Yu, Min Shi, Tessandra Stewart, Yang Huang, Zongran Liu, Guoyu Lan, Lifu Sheng, Chen Tian, and Dishun Yang contributed to the data acquisition and analysis. Zhenwei Yu Robin Barry Chan, Min Shi, and Jing Zhang contributed to drafting the manuscript and figures.

## CONFLICT OF INTEREST STATEMENT

Nothing to report.

## Supporting information


Figure S1.

Figure S2.

Table S1.

## Data Availability

The data that support the findings of this study are available on request from the corresponding author. The data are not publicly available due to privacy or ethical restrictions.
