# Supplementary figures and images for: GV‐971 attenuates α‐Synuclein aggregation and related pathology
Source: CNS Neurosci Ther. 2023 Aug 10;30(2):e14393. doi: 10.1111/cns.14393 (PMC10848097; doi:10.1111/cns.14393)

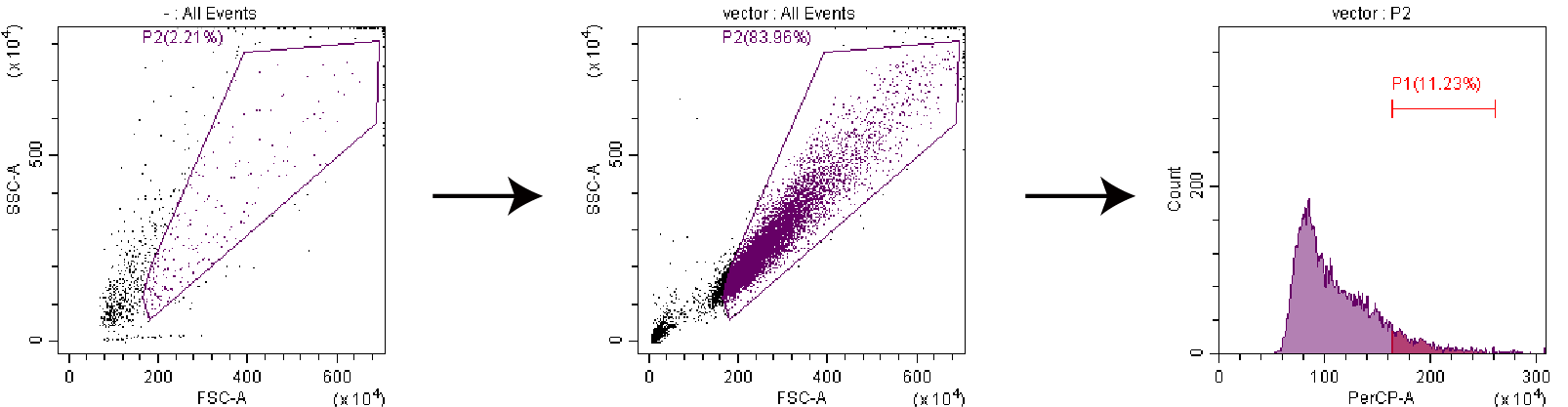

Supplement: Supplementary file 1 — Figure S1. [file CNS-30-e14393-s003.tif]

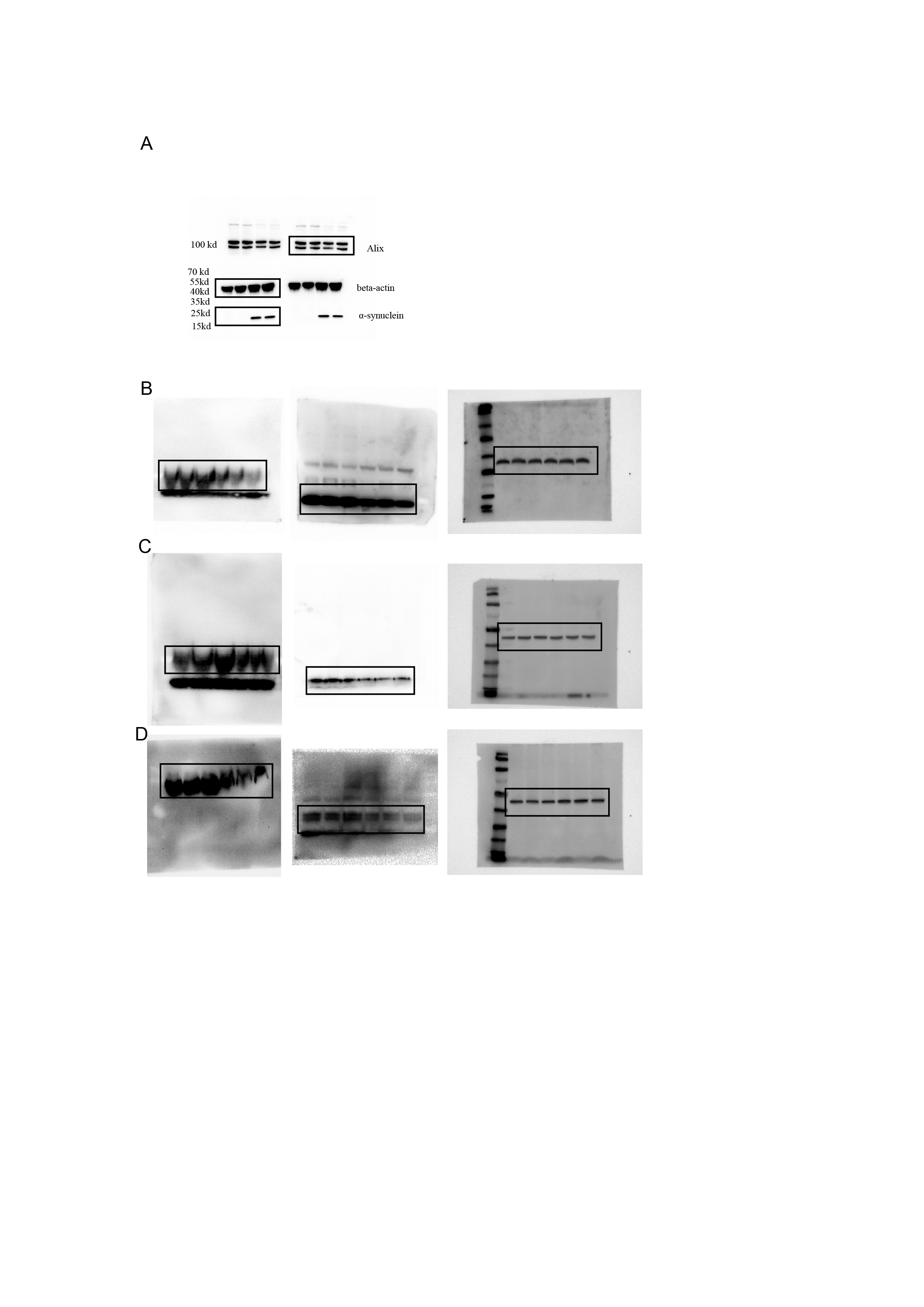

Supplement: Supplementary file 2 — Figure S2. [file CNS-30-e14393-s001.tif]
