# Supplementary material for: GV‐971 attenuates α‐Synuclein aggregation and related pathology
Source: CNS Neurosci Ther. 2023 Aug 10;30(2):e14393. doi: 10.1111/cns.14393 (PMC10848097; doi:10.1111/cns.14393)
Supplement: Supplementary file 3 — Table S1. [file CNS-30-e14393-s002.docx]

| **Supplementary Table 1 Demographic information** | | | |
| --- | --- | --- | --- |
| Diagnosis | PD | DLB | HC |
| Number | 5 | 5 | 5 |
| Age (mean +- SD) | 84.6 +- 6.65 | 84 +- 5.34 | 81 +- 4.85 |
| Sex (male: female) | 3: 2 | 3: 2 | 3: 2 |
